# Evaluating polymer interplay after hot water pretreatment to investigate maize stem internode recalcitrance

**DOI:** 10.1186/s13068-021-02015-8

**Published:** 2021-07-31

**Authors:** Amandine Leroy, Xavier Falourd, Loïc Foucat, Valérie Méchin, Fabienne Guillon, Gabriel Paës

**Affiliations:** 1 INRAE, UR 1268 BIA, 44316 Nantes, France; 2 INRAE, BIBS Facility, 44316 Nantes, France; 3 INRAE, Institut Jean-Pierre Bourgin, 78026 Versailles, France; 4 Université de Reims Champagne Ardenne, INRAE, FARE, UMR A614, 51100 Reims, France

**Keywords:** Hot water pretreatment, Maize, Multi-scale, NMR, Ultrastructure, Water mobility, Interactions, Accessibility

## Abstract

**Background:**

Biomass recalcitrance is governed by various molecular and structural factors but the interplay between these multiscale factors remains unclear. In this study, hot water pretreatment (HWP) was applied to maize stem internodes to highlight the impact of the ultrastructure of the polymers and their interactions on the accessibility and recalcitrance of the lignocellulosic biomass. The impact of HWP was analysed at different scales, from the polymer ultrastructure or water mobility to the cell wall organisation by combining complementary compositional, spectral and NMR analyses.

**Results:**

HWP increased the kinetics and yield of saccharification. Chemical characterisation showed that HWP altered cell wall composition with a loss of hemicelluloses (up to 45% in the 40-min HWP) and of ferulic acid cross-linking associated with lignin enrichment. The lignin structure was also altered (up to 35% reduction in β–O–4 bonds), associated with slight depolymerisation/repolymerisation depending on the length of treatment. The increase in ${T}_{1\rho }^{H}$, ${T}_{HH}$ and specific surface area (SSA) showed that the cellulose environment was looser after pretreatment. These changes were linked to the increased accessibility of more constrained water to the cellulose in the 5–15 nm pore size range.

**Conclusion:**

The loss of hemicelluloses and changes in polymer structural features caused by HWP led to reorganisation of the lignocellulose matrix. These modifications increased the SSA and redistributed the water thereby increasing the accessibility of cellulases and enhancing hydrolysis. Interestingly, lignin content did not have a negative impact on enzymatic hydrolysis but a higher lignin condensed state appeared to promote saccharification. The environment and organisation of lignin is thus more important than its concentration in explaining cellulose accessibility. Elucidating the interactions between polymers is the key to understanding LB recalcitrance and to identifying the best severity conditions to optimise HWP in sustainable biorefineries.

**Supplementary Information:**

The online version contains supplementary material available at 10.1186/s13068-021-02015-8.

## Highlights

- Reorganisation of the lignocellulosic matrix after water was redistributed by HWP
- Lignin reorganisation plus increased condensation promoted enzymatic deconstruction
- Multiscale approach combining chemical, spectral and NMR analyses helped elucidate polymer interplay.

**Supplementary Information:**

The online version contains supplementary material available at 10.1186/s13068-021-02015-8.

## Introduction

Valorisation of lignocellulosic biomass (LB) is a promising way to develop a sustainable bioeconomy thereby reducing our carbon footprint on the environment [1]. Each year, more than 1.3 billion tons of LB is generated worldwide and only 3% is valorised [1, 2]. The high energy content is the main interest of LB, meaning it will be economically competitive with fossil resources in the long term [3]. More than 90% of LB is composed of three highly connected polymers: cellulose, hemicelluloses, and lignin. In the biorefinery concept, pretreatment, enzymatic hydrolysis and fermentation steps can convert these polymers into bioproducts including high added value molecules, materials or energy [3–5]. Despite its high potential, valorisation of LB is still limited by its recalcitrance against hydrolytic deconstruction by enzymes, hampering the release of fermentable sugars, the precursors of biosourced products [6, 7].

Biomass accessibility is considered to be the main factor responsible for recalcitrance; it is influenced by various factors specific to LB which are difficult to apprehend due to their strong interconnections [7–9]. Among the factors which contribute to recalcitrance, those linked to the composition of the LB, such as hemicellulose or lignin content, can be distinguished from structural factors, including porosity, specific surface area of the cellulose, and ultrastructural factors related to cellulose (crystallinity, degree of polymerisation) [7]. The goal of pretreatment is precisely to reduce LB recalcitrance by “opening” the polymer network thereby improving the enzymes’ access to LB, notably to cellulose [4, 10]. Different pretreatments have been developed to break down and restructure the lignocellulosic matrix [8, 11, 12]. These pretreatments fall into four categories: physical (milling, ultra sound, etc.), chemical (ionic liquid, dilute acid, organosolv, etc.), physico-chemical (steam explosion, hot water, oxidative, etc.) and biological (fungi, bacteria or archaea) pretreatments [11, 13, 14]. Among the physico-chemical pretreatments, hot water pretreatment (HWP), also called hydrothermal pretreatment or autohydrolysis, stands out [15–17] as it only uses hot water as catalyst, generally at temperatures ranging from 170 to 230 °C. The catalyst is kept in the liquid state by applying high pressure, up to 5 MPa at the highest temperatures [13, 18]. The absence of corrosive compounds makes HWP one of the most economical, easy to use and environmentally friendly pretreatments [18].

The HWP of LB has been extensively investigated to determine its effect and to advance our understanding of the factors that contribute to LB recalcitrance. Most frequently, the effect of HWP is analysed based on variations in the chemical composition of the LB, more specifically that of the three major polymers. It is now well known that hemicelluloses is the main polysaccharide impacted by HWP [17, 19–22]. Indeed, due to their amorphous structure, hemicelluloses are the components most susceptible to hydrolysis through breakage of glycosidic bonds by the organic acids generated during the pretreatment [3, 18]. HWP also changes other lignocellulosic components, including lignin and hydroxycinnamic acids, by breaking the inter- and intra-polymer bonds involving ferulic acid molecules [18, 23, 24], the depolymerization and the repolymerization of lignin as a drop on the cell wall surface [21, 25–27]. Through their direct impact on the structural parameters of the lignocellulosic matrix, these chemical modifications have been shown to improve the deconstruction capacity of the enzymes. Three scales of structural modifications by HWP can be used: cell wall porosity (associated with the mobility of enzymes), interactions, and polymer ultrastructure [28–30]. Several methods have been developed to study the porosity of the LB using different probes: polymers in solute exclusion [31], water molecules in NMR relaxometry (low-field NMR) or mercury in porosimetry [32–34]. Other techniques are used to obtain an indirect estimation of porosity, such as Simons’ staining, using dyes with a high affinity for cellulose, which also make it possible to estimate the accessible surface area of cellulose for adsorption of cellulases during enzymatic hydrolysis [35]. These techniques highlight the modifications in pore volume and pore size after the pretreatment, thereby increasing enzyme accessibility and subsequently also increasing the saccharification yield [32, 33, 36–38]. The impact of HWP on the ultrastructure of cellulose, via an increase in its degree of crystallisation and a decrease in its degree of polymerisation, is well known but relatively few studies have analysed the environment and the interaction capacity of the cellulose. Information on the cellulose environment and how it interacts with the other components of LB can be obtained via solid-state NMR and has led to progress in understanding polymer interactions [39, 40]. However, no studies have exploited the potential of this method to analyse changes in polymer composition and organisation within the cell wall following the application of HWP. Despite its importance, the interplay between the chemical and structural factors which control HWP and contribute to recalcitrance is still not understood.

In this study, the impact of HWP on the different factors associated with LB recalcitrance was examined using complementary methods that probe different structural levels. Simons’ staining, solid-state NMR and low-field NMR analyses were combined to assess porosity and water–biomass interactions and to characterise the structure and the environment of the cellulose. The variation of the multi-scale factors was correlated with saccharification to advance our understanding of the impact of physico-chemical parameters on the accessibility of LB. Due to their agricultural and economic importance, maize residues were chosen as a model of grass LB.

## Results and discussion

### Influence of HWP on saccharification profiles

**Fig. 1:**
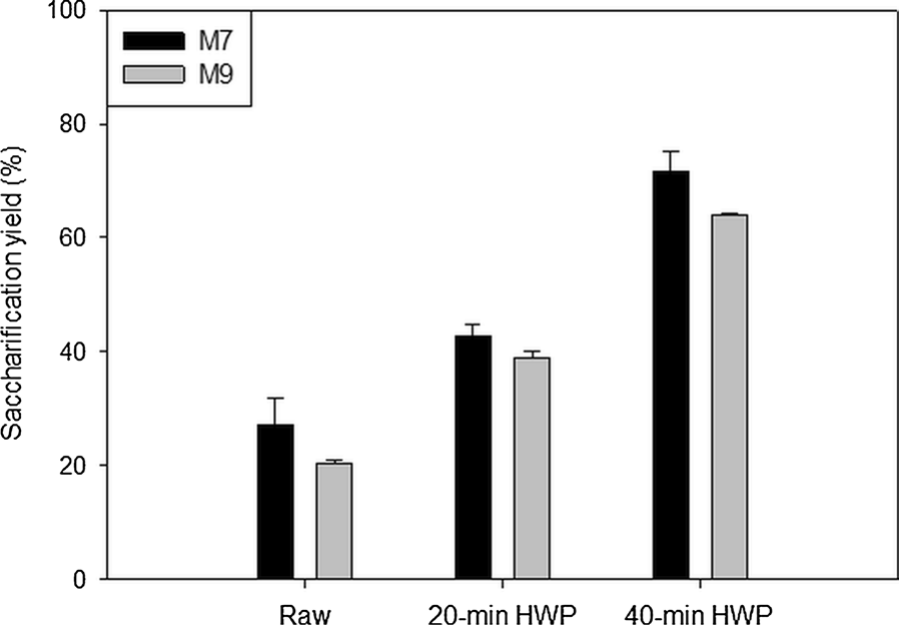
Saccharification yield of raw and HWP samples after 72 h

Two maize genotypes F7025 (hereafter M7) and F98902 (hereafter M9) were used for this study. HWP increased the conversion of sugars in both genotypes, and improvement increased with an increase in the duration of the pretreatment (Fig. 1). The percentage of sugars converted during saccharification increased 1.6 to 3.1 fold compared to the raw material, depending on the genotype and on the duration of HWP, in agreement with results reported in the literature on corn straw [41] or in other grass species including sugarcane [15, 42], wheat and miscanthus [33]. The initial reaction rates also increased 1.2 and 2.4 fold in M7 samples and 2.2 and 3.6 fold in M9 samples when the pretreatment time was increased from 20 to 40 min (Additional file 1). These observations show that HWP increases the accessibility and hydrolysis capacity of the hydrolytic enzymes, as described below.

### Changes in the chemical composition and structure of cell wall polymers

#### Changes in chemical composition

The changes in composition which occurred upon HWP were first determined by FTIR spectra combined with chemical analyses.

**Fig. 2:**
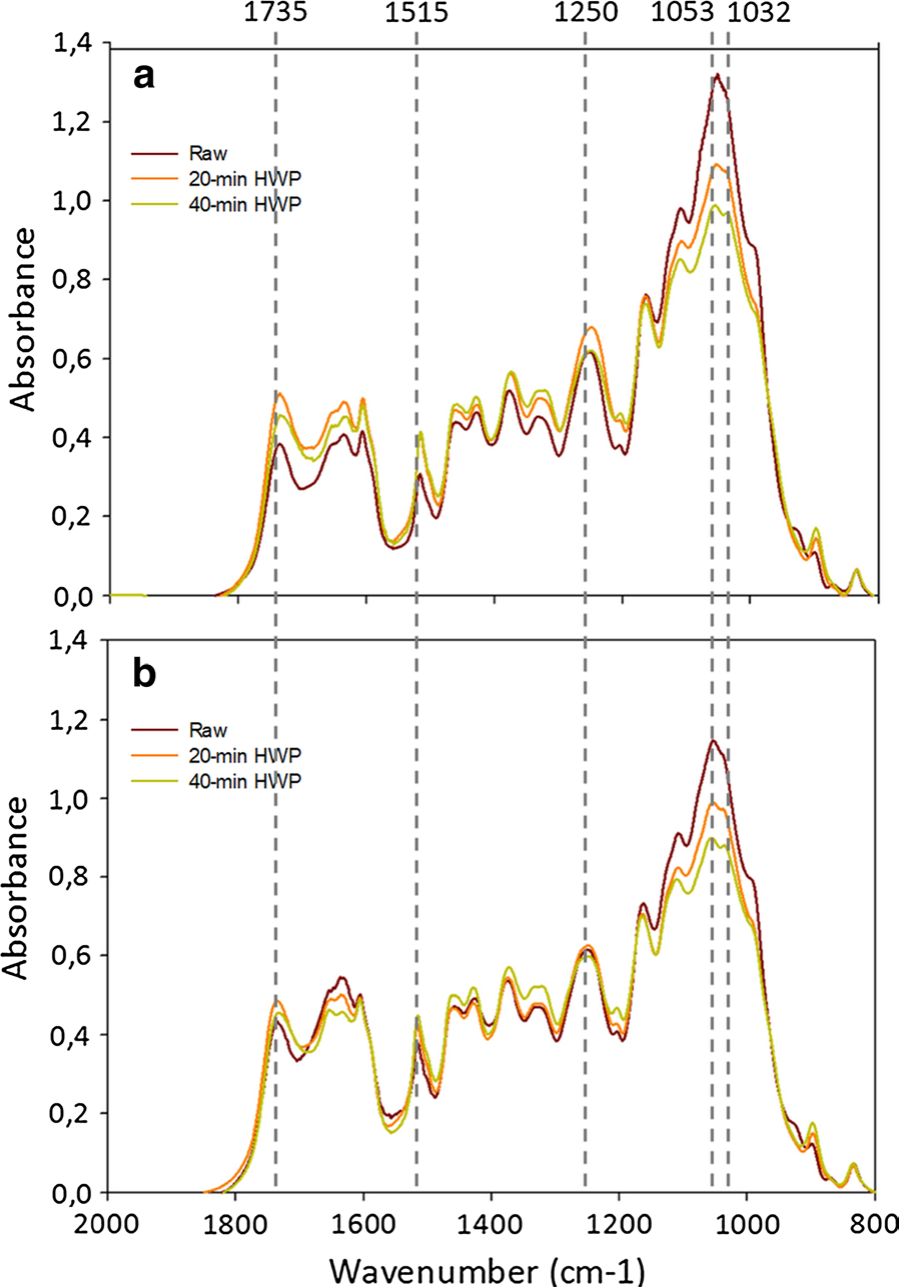
FTIR spectra of raw and pretreated **a** M7 and **b** M9 samples

The two genotypes had differing absorbance profiles in the spectral region from 800 to 1200 cm^−1^, corresponding to the region of polysaccharide absorption [43] and at 1515 cm^−1^, characteristic of C=C stretching of lignins (Fig. 2). Absorbance in the sugar region was higher in the M7 genotype, pointing to higher sugar content in this genotype. In contrast, the peaks characteristic of lignin were higher in the M9 genotype, revealing enrichment in lignin in this genotype.

Following HWP, a decrease in the absorption was observed between 800 and 1200 cm^−1^ (Fig. 2). Absorbance at 1053 cm^−1^, which is typical of the C–O stretching band of linear or branched β-(1,4) xylans, decreased with an increase in HWP time. This likely corresponds to a decrease in glucuronoarabinoxylan (GAX) content in the CWR [44–46]. The decrease in GAX content resulted in enrichment in cellulose and lignin, as shown by the appearance of the peak at 1032 cm^−1^, corresponding to the C–O stretching signal of β-(1-4) glucans, and an increase in the peak at 1515 cm^−1^. The variation of non-conjugated esters from polysaccharide components was shown at wavelengths 1735 cm^−1^ and 1250 cm^−1^ [47]. Interestingly, the absorbance of these bands increased with a 20-min HWP and subsequently decreased with a 40-min HWP in both genotypes.

The polysaccharide content, lignin content and the cellulose/hemicelluloses ratio in raw M7 and M9 CWR were 60.2/67.3%, 16.3/18.6% and 2.09/1.64, respectively (Table 1). For hemicelluloses, ^13^C CP/MAS NMR analyses showed that the molar proportion of hemicelluloses, expressed relative to the sum of hemicelluloses and cellulose, remained stable after a 20-min HWP and decreased by, respectively, 6% and 4% after a 40-min HWP (Table 2). The non-reduction of the molar proportion of hemicellulose after the 20-min pretreatment may be related to an equivalent molar loss of cellulose at this pretreatment severity. Quantification of sugars by HPAEC-PAD (Table 1) revealed a decrease in the relative hemicellulose content according to the duration of the HWP. This decrease corresponded to a GAX solubilization of 13.4% and 50.5% for the M7 and of 19.6% and 45.7% for the M9 genotype compared to the GAX content in the raw materials for the HWP 20 min and HWP 40 min, respectively. During the HWP, the hemicellulose fraction was removed by hydrolysis of the glycosidic bonds in the xylan backbone via hydronium ions and organic acids produced by auto-ionization of water and the release of organic acids (acetic acid) and uronic acids [18, 48].

**Table 1:** Composition of CWR and percentage of solubilized or lost macromolecular fractions after the HWP

	Raw maize stem		20-min HWP		40-min HWP	
	M7	M9	M7	M9	M7	M9
Pretreatment severity	–	–	3.66	3.66	3.96	3.96
Yield of HWP (g g^−1^ CWR)	-	–	0.797 (± 0.022)	0.796 (± 0.034)	0.629 (± 0.027)	0.636 (± 0.033)
Hydrolysate pH	–	–	4.4 (± 0.06)	4.4 (± 0.08)	3.8 (± 0.01)	3.8 (± 0.06)
Composition						
Polysaccharides (g 100 g^−1^ CWR)	60.2 (± 0.7)	67.3 (± 1.5)	65.7 (± 1.9)	62.1 (± 4.4)	65.1 (± 2.6)	64.0 (± 0.3)
Cellulose (g.100 g^−1^ of sugars)	67.7 (± 0.1)	62.1 (± 0.4)	58.4 (± 0.1)	62.4 (± 0.3)	76.7 (± 0.3)	69.2 (± 0.8)
Hemicelluloses (g.100 g^−1^ of sugars)	32.3 (± 0.1)	37.9 (± 0.4)	41.6 (± 0.1)	37.6 (± 0.3)	23.3 (± 0.3)	30.8 (± 0.8)
Klason lignin (g.100 g^−1^ CWR)	16.3 (± 0.1)	18.6 (± 0.4)	18.7 (± 0.2)	20.1 (± 0.3)	25.3 (± 1.3)	27.3 (± 0.2)
Loss/solubilization after HWP^a^						
Cellulose (%)	–	–	25.3 (± 1.5)	23.9 (± 5.3)	23.1 (± 1.0)	30.6 (± 0.8)
Hemicelluloses (%)	–	–	13.4 (± 3.2)	19.6 (± 4.2)	50.5 (± 3.4)	45.7 (± 1.1)
Klason lignin (%)	–	–	8.7 (± 1.0)	11.8 (± 1.1)	3 (± 0.4)	6.6 (± 0.7)

Results are expressed as means of three repetitions with standard deviation in brackets

^a^ Loss/solubilization after HWP (%) = 100 × [1 − *MY* × (*Y*_*f*_/*Y*_*i*_)], where MY is the mass yield of the HWP, Y_f_ is the final content and Y_i_ is the initial content (raw CWR)

**Table 2:** CP/MAS ^13^C NMR analysis of the molar proportion of hemicellulose

	Raw maize stem		20-min HWP		40-min HWP	
	M7	M9	M7	M9	M7	M9
Hemicellulose molar proportion (%)	21 (± 2)	20 (± 1)	20 (± 1)	19 (± 1)	15 (± 0)	16 (± 2)

Results are expressed as means of three repetitions with standard deviation in brackets

In contrast, HWP led to up to 11.7% and 10.3% increase in cellulose in the 40-min HWP in genotypes M7 and M9, respectively. Solubilization of the cellulose ranging from 23.1 to 30.6% was detected with HWP. This loss was slightly higher than that reported in another study on corn stover [41] (between 4 and 22% [18, 49]).

The relative lignin content increased by 7.5–13% after the 20-min HWP and by between 31.8% and 35.5% after the 40-min HWP, depending on the genotype. A minor loss of lignin was observed in HWP CWR ranging from 3% to 11.8%. Lignin solubilization was more pronounced with the 20-min HWP, suggesting depolymerization/repolymerization of lignin according to the length of the HWP due to acidification of the medium leading to the breaking of carbohydrate–lignin bonds [25, 48, 50].

These analyses showed that the HWP altered the relative concentration of the three main polymers, with loss of hemicelluloses. This could have an impact on the composition and/or structure of the lignin–carbohydrate complex (LCC) and indirectly on the hydrolysis capacity [25].

#### Impact on phenolic compounds

Grasses are rich in hydroxycinnamic acid ester- and ether-bounded, mainly ferulic acid (FA) and *p*-coumaric acid (*p*-CA) [51, 52]. The concentrations of FA and *p*-CA ether- and ester-bounded were thus determined (Table 3).

**Table 3:** Ester- and ether-linked hydroxycinnamic acid content in raw and HWP CWR

	Raw maize stem		20-min HWP		40-min HWP	
	M7	M9	M7	M9	M7	M9
*p*-coumaric acid (g.100 g^−1^ CWR)						
Ester linked	2.58 (± 0.4)	2.20 (± 0.6)	2.87 (± 0.2)	2.66 (± 0.7)	2.76 (± 0.2)	2.31 (± 0.1)
Ferulic acid (g 100 g^−1^ CWR)						
Ester linked	0.55 (± 0.01)	0.50 (± 0.01)	0.71 (± 0.01)	0.60 (± 0.02)	0.42 (± 0.01)	0.34 (± 0.01)
Diferulic acid ester linked	0.11 (± 0.01)	0.09 (± 0.01)	0.15 (± 0.01)	0.09 (± 0.01)	0.02 (± 0.01)	0.02 (± 0.01)
Ether linked	0.44	0.42	0.27	0.34	0.37	0.39

Results are expressed as means of three repetitions with standard deviation in brackets

*p*-CA is mainly linked to the side chain of the lignin syringyl units and, to a lesser extent, to the GAX arabinose units [53]. The *p*-CA content in the raw and HWP CWR remained stable, representing between 2.2% and 2.8% of their CWR. Despite an enrichment in lignin, the stable *p*-CA suggests that HWP induced homogeneous *p*-CA solubilization according to the mass loss ranging from 4 to 11% in the 20-min HWP and 33% in the 40-min HWP.

Phenolic compounds in the cell wall are closely bound to carbohydrates through numerous bonds forming LCCs. In grasses, ferulic acid is the cross linking agent which mainly enables the formation of these complexes by forming GAX–GAX bonds via esterified diferulic acids (DiFAe) on arabinose units and GAX–lignin bonds via ether bonds on guaiacyl units of lignin [53–55]. Numerous studies have shown that the complex network formed by the cross linking of FAs between hemicelluloses and lignin reduces polysaccharide deconstruction by limiting access to the enzymes [56].

In the raw genotypes, 60% of FA units were found to be linked to arabinose, while 40% were involved in ether linkages with lignin. DiFAe content, which was linked to the number of hemicellulose bonds, decreased significantly by more than 88% after the 40-min HWP. Interestingly, with the 20-min HWP, the amount of DiFAe binding remained stable in the M7 CWR and decreased by 19% in the M9 CWR. This difference between the two genotypes shows that the 20-min HWP allows more efficient disruption of the hemicellulosic network in M9, which is consistent with the higher hemicellulose loss in this genotype.

The concentration of GAX–lignin bonds was estimated using the content of ether linked FA. In contrast to the GAX–GAX bonds, a significant number of AX–lignin bonds were broken after 20 min of the HWP. As observed for lignins, the loss of etherified FA was higher after the 20-min HWP with 50/36% loss than after the 40-min HWP with 46/40% in M7 and M9, respectively. This likely originates from depolymerization/re-polymerization or reorganisation of the lignins during the pretreatment. In addition, these results suggest that HWP causes sequential changes in the LCC with a prior step of GAX–lignin disruption followed by GAX–GAX perturbations, as already suggested [57]. This hypothesis is strengthened by the low loss of hemicelluloses and the higher loss of lignin with a 20-min HWP, showing prior disruption of the lignin fraction. However, it should be noted that the amount of FA engaged in ether bonds with lignin may be underestimated due to the complexity of the lignin which may prevent the hydrolysis of total ether linked FA.

#### Changes in the polymer ultrastructure

To assess the influence of the parameters specific to the composition of the lignocellulose on the structural modifications, a correlation matrix was carried out and is presented in Additional file 2.

##### Lignin

As shown by the hydroxycinnamic acids and lignin loss, the HWP had an impact on the organisation of the lignin depending on the severity of the pretreatment. The fluorescence intensity of the CWR was measured relative to the overall structural properties of the lignin (Table 4). A 42–53% and 79% decrease in fluorescence intensity was detected for the 20-min HWP and 40-min HWP, respectively, compared to raw CWR. Given that the intensity of fluorescence of the CWR depends on the composition of the lignin, on the type of bonds between the monolignols and lignin environment [58], this result suggests HWP causes a general structural/compositional change in the lignin fraction.

**Table 4:** Lignin characteristics: thioacidolysis yield, S/G ratio and fluorescence intensity

	Raw maize stem		20-min HWP		40-min HWP	
	M7	M9	M7	M9	M7	M9
Thioacidolysis yield (µmol g^−1^ KL)	765 (± 23)	671 (± 43)	799 (± 11)	884 (± 12)	321 (± 33)	266 (± 8)
S/G ratio	1.79 (± 0.02)	1.02 (± 0.03)	1.96 (± 0.14)	0.99 (± 0.01)	2.87 (± 0.10)	1.35 (± 0.10)
Fluorescence intensity	8100	5500	4700	2600	1700	1200

Thioacidolysis yields are expressed in µmol g^−1^.Klason Lignin. Syringyl/Guaiacyl molar ratios (S/G ratio) were determined by thioacidolysis. Fluorescence intensity was measured at λ_excitation_ = 365 nm and λ_emission_ = 440 nm. Results are expressed as means of three repetitions with standard deviation in brackets

The ultrastructural modification of the lignin was investigated in more detail by analysing the thioacidolysis yield and the ratio of syringyl to guaiacyl lignin units (S/G) (Table 4). The thioacidolysis yield and S/G ratio were 764/672 µmol g^−1^ of Klason lignin and 1.79/1.02 in raw M7 and M9, respectively: this means that M7 had higher β–O–4 bond and S unit contents. Even if the loss of lignin induced by HWP was limited (less than 12%), changes in the lignin structure did occur. Indeed, based only on the cleavage of non-condensed ether β–O–4 bonds which are predominant in grasses [59], the yield of thioacidolysis decreased significantly, by 58% and 60%, with the 20-min HWP and the 40-min HWP in M7 and M9, respectively. The S/G ratio was gradually impacted by HWP with an increase as a function of severity. The number of G units decreased with the 40-min pretreatment. This suggests that the β–O–4 bonds of the G units, which are determined by thioacidolysis, were more affected by HWP and, therefore, more susceptible to condensation than the S units, as already shown in rice and sugarcane bagasse [60, 61].

^13^C CP/MAS NMR was also performed to estimate the abundance of β–O–4 bonds representative of the entire lignin polymer [62]. For this purpose, the area of the two broad signals were measured at 152.4 ppm which may be associated with the aromatic carbon atoms of lignin involved in β–O–4 bonds, such as C-3 and C-5 of the S units, and at 146.8 ppm, corresponding to the same carbons non-etherified (Table 5). Depletion of the β–O–4 bonds was detected with the HWP and represented a decrease of 17% and 25% in M7, and of 12% and 35% in M9 with the 20-min and 40-min HWP, respectively. This depletion, highlighting condensation of the lignin caused by the acidification of the medium during pretreatment, has also been reported in other grass species as well as in hardwood with HWP and dilute acid pretreatments [33, 63–67].

**Table 5:** CP/MAS and VCT ^13^C NMR analysis of raw and pretreated CWR

	Raw maize stem		20-min HWP		40-min HWP	
	M7	M9	M7	M9	M7	M9
Crystallinity (%)	28 (± 2)	31 (± 1)	32 (± 1)	34 (± 2)	39 (± 1)	41 (± 1)
LFD (nm)	2.4 (± 0.1)	2.6 (± 0.1)	2.6 (± 0.1)	2.7 (± 0.1)	3.0 (± 0.1)	3.1 (± 0.1)
LFAD (nm)	25 (± 3)	27 (± 3)	39 (± 5)	31 (± 9)	37 (± 5)	74 (± 4)
Estimated β–O–4 bonds (%)	48 (± 1)	49 (± 2)	40 (± 3)	43 (± 1)	36 (± 1)	32 (± 2)
${T}_{1\rho }^{H}$ (ms)	18 (± 0)	22 (± 3)	33 (± 3)	37 (± 6)	45 (± 6)	50 (± 9)
${T}_{HH}$ (µs)	416 (± 32)	396 (± 61)	450 (± 73)	458 (± 100)	671 (± 121)	597 (± 122)

Lateral fibre dimension (LFD), lateral fibre aggregate dimension (LFAD), spin–lattice (proton relaxation) in the rotating frame (${T}_{1\rho }^{H}$) and time of spin diffusion (${T}_{HH}$). The analysis was conducted on rehydrated CWR (20% w/w) and the results are expressed as means of three repetitions with standard deviation in brackets

Overall, HWP altered the ultrastructure of the lignin by breaking the β–O–4 bonds via the acidic medium of the pretreatment. These modifications led to changes in the structure of the lignin surrounding the cellulose, which could also have an impact on the ultrastructure of the cellulose.

##### Cellulose

**Fig. 3:**
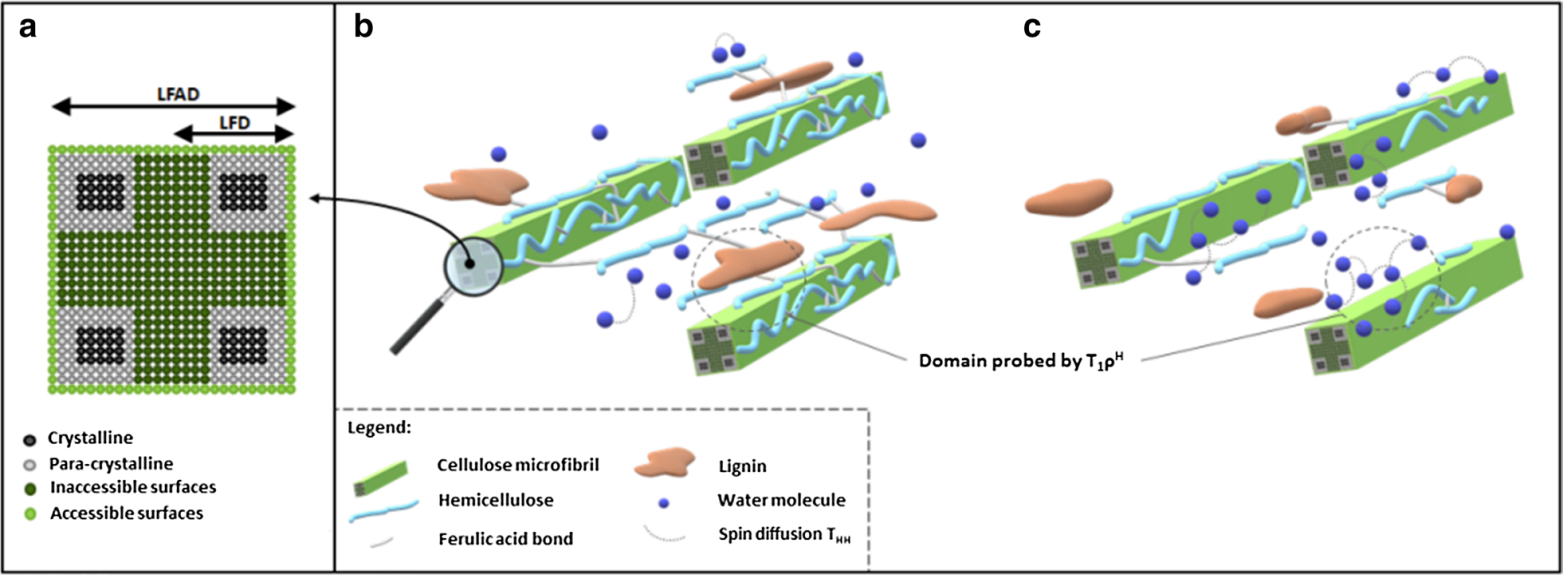
Schematic representation of raw cellulose fibril and cell wall organisation. **a** Schematic representation of raw cellulose fibril with the representation of the four cellulose forms and the two fibril aggregate dimensions: lateral fibril dimension (LFD), and lateral fibril aggregate dimensions (LFAD) (Adapted from [69]); Schematic representation of cellulose microfibrils distribution within lignocellulosic matrix and of the ${T}_{1\rho }^{H}$ and ${T}_{HH}$ variations of **b** raw sample and **c** HWP sample according to the hemicellulose loss and lignin condensation

Like for lignin, ^13^C CP/MAS NMR was performed to determine the impact of the HWP on the ultrastructure of cellulose. For this purpose, crystallinity, and fibril dimensions, through lateral dimension of the microfibrils (LFD) and of the microfibril aggregates (LFAD), were determined by spectral deconvolution of the region of the cellulose C4 signals at 80–91 ppm (Table 4). LFD and LFAD were determined using a conversion factor of 0.57 nm per cellulose chain, and are schematically represented in Fig. 3 [68, 69].

Both raw CWR had a similar cellulose ultrastructure with average crystallinity, LFD and LFAD values of about 30%, 2.5 nm and 26 nm, respectively, of around 26% at both 2.5 and 30 nm. The crystallinity of the CWR increased with increased severity of the HWP. This relative increase in crystallinity was linked to preferential degradation/solubilization of amorphous cellulose during pretreatment by breaking intra-chain hydrogen bonds. The solubilisation of amorphous cellulose likely explains some of the cellulose loss that occurred during the HWP shown above (Table 1). Studies comparing the effect of different types of pretreatments on crystallinity reported that the acidity of the pretreatment medium was directly linked with the solubilization of amorphous cellulose [65, 70]. During the HWP, the reaction medium was gradually acidified with an increase in pretreatment time, from a pH value of 4.4 at 20 min to 3.8 after the 40-min HWP (Table 1). Acidification was negatively correlated (*R*^2^ = − 0.89) with the increasing loss of amorphous cellulose ranging from 13 to 17% with the 20-min HWP to 36% to 38% with the 40-min HWP in M9 and M7, respectively.

HWP also led to changes in the ultrastructure of the cellulose by slightly increasing the LFD. During the HWP, the loss of hemicelluloses (*R*^2^ = 0.95) and amorphous cellulose (*R*^2^ = 0.93) as well as the rearrangement of lignin, shown by the decrease in thioacidolysis yield (*R*^2^ = 0.85) and estimated β–O–4 bonds (*R*^2^ = 0.90), could be linked to loosening of the cellulose microfibrils and possible swelling of the cellulose [70, 71].

The increase in LFD was accompanied by an increase in LFAD. The increase in LFAD could be explained by the decrease in water–cellulose interactions (promoting cellulose–cellulose interactions) induced by heat, leading to an increase in the size of the aggregates [71–73]. The 40-min HWP significantly modified the LFAD of genotype M9 compared to genotype M7. This difference suggests a strong hornification effect in M9 with the 40-min HWP due to notable co-crystallization of the cellulose induced by an efficient relocalization/modifications of the lignins and loss of hemicellulose surrounding the cellulose and acting as spacer [36, 74–76].

The HWP led to changes in the cellulose architecture, with an increase in the dimension of the microfibrils and their aggregates. These changes in the structure of the cellulose suggest changes in the interactions between cellulose and its environment due to the pretreatment.

### Changes in interactions and in entanglement in the cell wall

#### Changes in cellulose interactions

Chemical analyses showed that the HWP caused significant changes in the composition of LB, particularly by removing hemicellulose and by reorganizing the lignocellulosic components surrounding the cellulose, thereby modifying its accessibility. We, therefore, investigated the impact of HWP on the interactions and the accessibility of cellulose using solid-state NMR and by studying the kinetics of polarization transfer (Table 5).

First, the time constant ${T}_{1\rho }^{H}$ provided information about the molecular organization surrounding the cellulose, the longer the ${T}_{1\rho }^{H}$, the higher the molecular order [39]. An increase in ${T}_{1\rho }^{H}$ was observed in both M7 and M9 with an increase in the pretreatment time from 17/21 ms for raw CWR to 43/51 ms for the most severely pretreated CWR. This increase was positively correlated with crystallinity (*R*^2^ = 0.97) and with hemicellulose loss (*R*^2^ = 0.95), suggesting an increase in the molecular order surrounding the cellulose microfibrils after HWP due to the removal of amorphous components, cellulose, and hemicelluloses (Fig. 3). These results are in agreement with those of [40] and [77], who reported an increase in ${T}_{1\rho }^{H}$ in wheat with HWP and in poplar pretreated with dilute acid, respectively.

The increase in molecular order was accompanied by an increase in the proton spin diffusion time (${T}_{HH}$) after HWP in both genotypes. ${T}_{HH}$ reflects the diffusion of non-bounded proton magnetisation, which generally corresponds to the protons of water, close to cellulose [78, 79]. The increase in ${T}_{HH}$ was positively correlated with the loss of hemicelluloses (*R*^2^ = 0.98) and DiFA content (*R*^2^ = 0.84), and with the concentration of Klason lignin (*R*^2^ = 0.91), and was negatively correlated with the proportion of S-units involved in ether bonds (*R*^2^ = − 0.86). This suggests that the increase in the molecular order due to the elimination of amorphous components, results in an increase in the spaces between the microfibrils, thereby facilitating access by the water molecules and their interactions with cellulose.

The HWP not only changed the ultrastructure of the cellulose but also its interactions with hemicelluloses/lignins, making the microfibrils more accessible to the water molecules. These modifications suggest changes in the porosity of the cell wall as well as in the accessible surface of the cellulose after HWP, thereby leading to an increase in the interactions between the celluloses and the water and in turn, with hydrolytic enzymes.

#### Porosity and accessibility of cellulose

Simons’ staining is widely used to estimate changes in the structure and in the environment of the cellulose caused by the pretreatment by analysing variations in relative porosity as well as in the total surface area of the cellulose accessible to cellulases [80]. Simons’ staining is based on the adsorption of two contrasted dyes: DB1 for small pores with a hydrodynamic radius (*r*_H_) of around 1 nm and DY11 for pore sizes larger than the *r*_H_ of cellulases [81, 82].

**Fig. 4:**
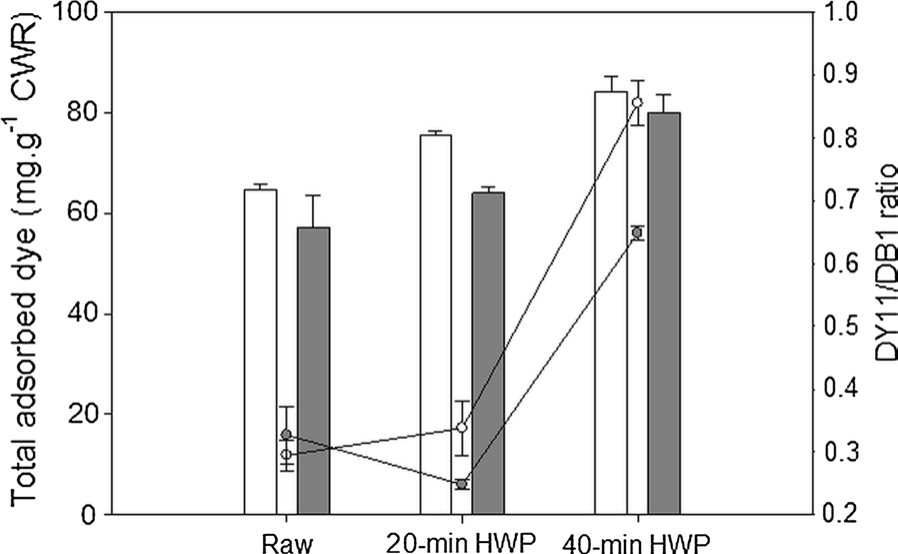
Changes in the total adsorbed dye and in the DY11/DB1 ratio of raw and pretreated samples. The total adsorbed dye are represented by bars with left axis and DY11/DB1 ratio by plots with the right axis. The M7 values are represented in white and M9 samples in gray

Variation in the total accessible surface area (ASA) of cellulose was estimated from the total amount of dyes adsorbed (Fig. 4). No significant differences in the total amount of probes adsorbed was observed between the two genotypes, in accordance with their similar environment and similar cellulose structure. An increase in the amount of dye adsorbed by the substrate was observed in both genotypes with increased HWP time, reflecting an increase in the ASA.

By measuring the total amount of adsorbed dyes and the DY11/DB1 ratio, it was possible to estimate the relative amount and size of the pores of the CWR after HWP [83]. Moreover, variation in the ratio alone provides information on the variation in the specific surface area (SSA) of the cellulose accessible to cellulases. In the raw genotypes, the close DY11/DB1 ratio pointed to an akin SSA between the two genotypes. After 20 min of HWP, the ratio differed with the genotype. In M7 after 20-min HWP, the increase in total adsorbed dye followed by a slight increase in the DY11/DB1 ratio, suggests the generation of new “small” pores and a slight expansion of the existing pores enabling an increase in the amount of adsorbed DB1 and DY11, respectively. In comparison, in M9, the 20-min HWP resulted in a decrease in the DY11/DB1 ratio, and in an increase in the total amount of adsorbed dye, which implies only the generation of “small” pores but no expansion of existing pores. When the HWP time was extended to 40 min, a significant increase in the DY11/DB1 ratio and in the amount of the total adsorbed dye was detected in both genotypes compared to in the raw CWR. These results prove that the 40-min HWP mainly resulted in the expansion of existing pores, enabling increased adsorption of DY11. The DY11/DB1 ratio values were 2.93 and 1.97 times higher than those of the raw CWR M7 and M9, respectively, showing a clear increase of SSA with this pretreatment time especially in M7.

Simons’ staining showed that the HWP both increased the relative number of pores and their expansion and also increased the SSA, two important parameters for the improvement of cellulose hydrolysis [30]. In the present study, the increase in the SSA induced by HWP was positively correlated with the increase in the diffusion time *T*_HH_ (*R*^2^ = 0.96) and the loss of hemicelluloses (*R*^2^ = 0.89) and amorphous cellulose (*R*^2^ = 0.88). This correlation shows that a higher water–cellulose interaction related to a higher molecular order increases the accessibility of cellulose to cellulases. It is worth noting that the increase in the SSA was negatively correlated with the yield of thioacidolysis (*R*^2^ = − 0.96), suggesting that the condensation of lignin caused by HWP facilitates access to cellulose by cellulases. Surprisingly, the increase in lignin content was positively correlated with the DY11/DB1 ratio (*R*^2^ = 0.86), indicating that lignin content does not necessarily contribute to recalcitrance and that changes in its structure could reduce its negative impact on cellulose accessibility. The effect of the HWP on the porosity of the cell wall that facilitates access to the cellulose was investigated in more detail using relaxometry analyses.

#### Water mobility

**Fig. 5:**
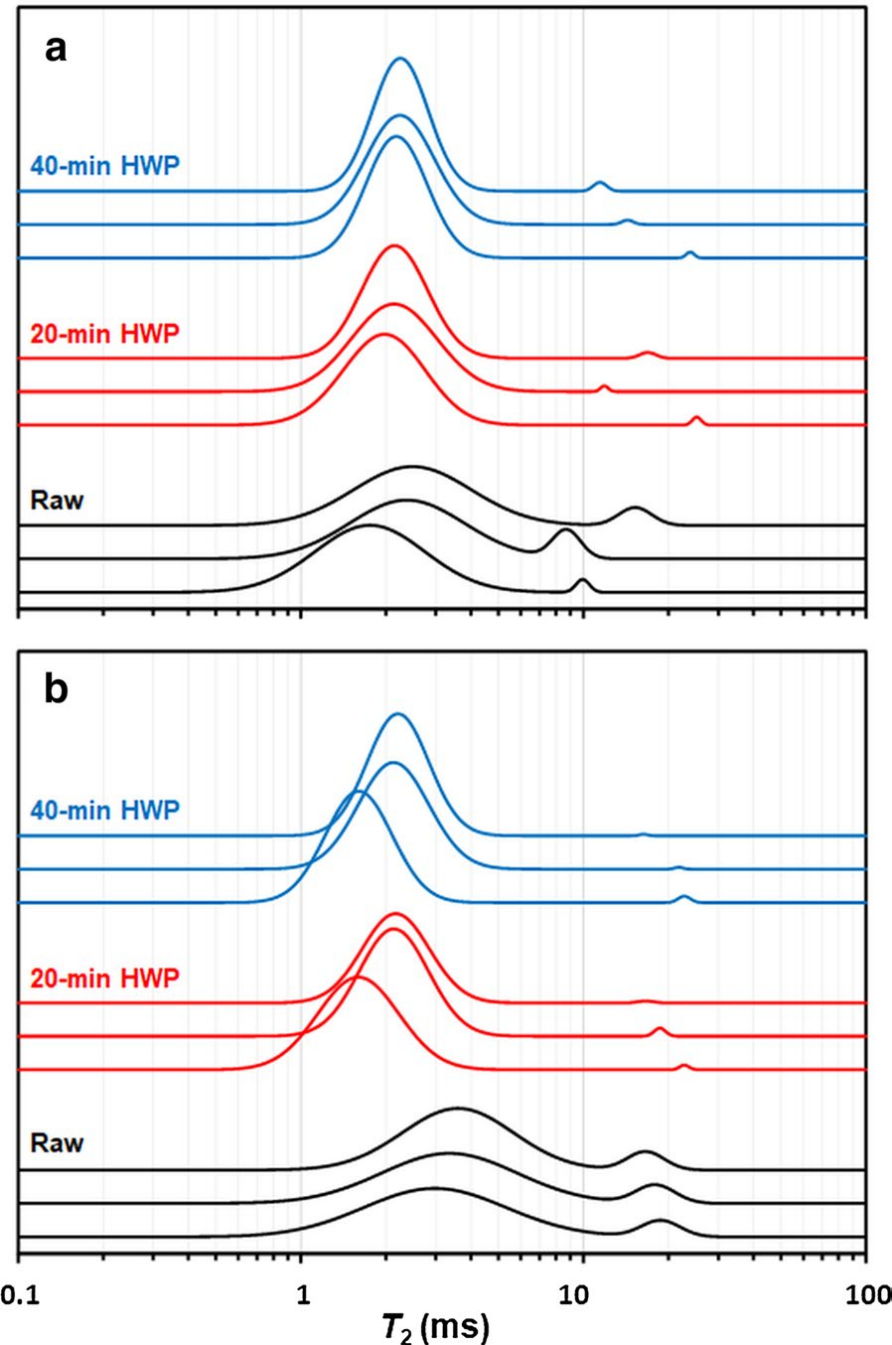
${T}_{2}$ distribution in **a** M7 and **b** M9 samples with a water content of 20% (w/w). The x axis is the logarithmic scale of the relaxation time. The Y axis corresponds to the distribution amplitude expressed in normalized relative water content (% w/w). The replicates are represented in the same color

**Fig. 6:**
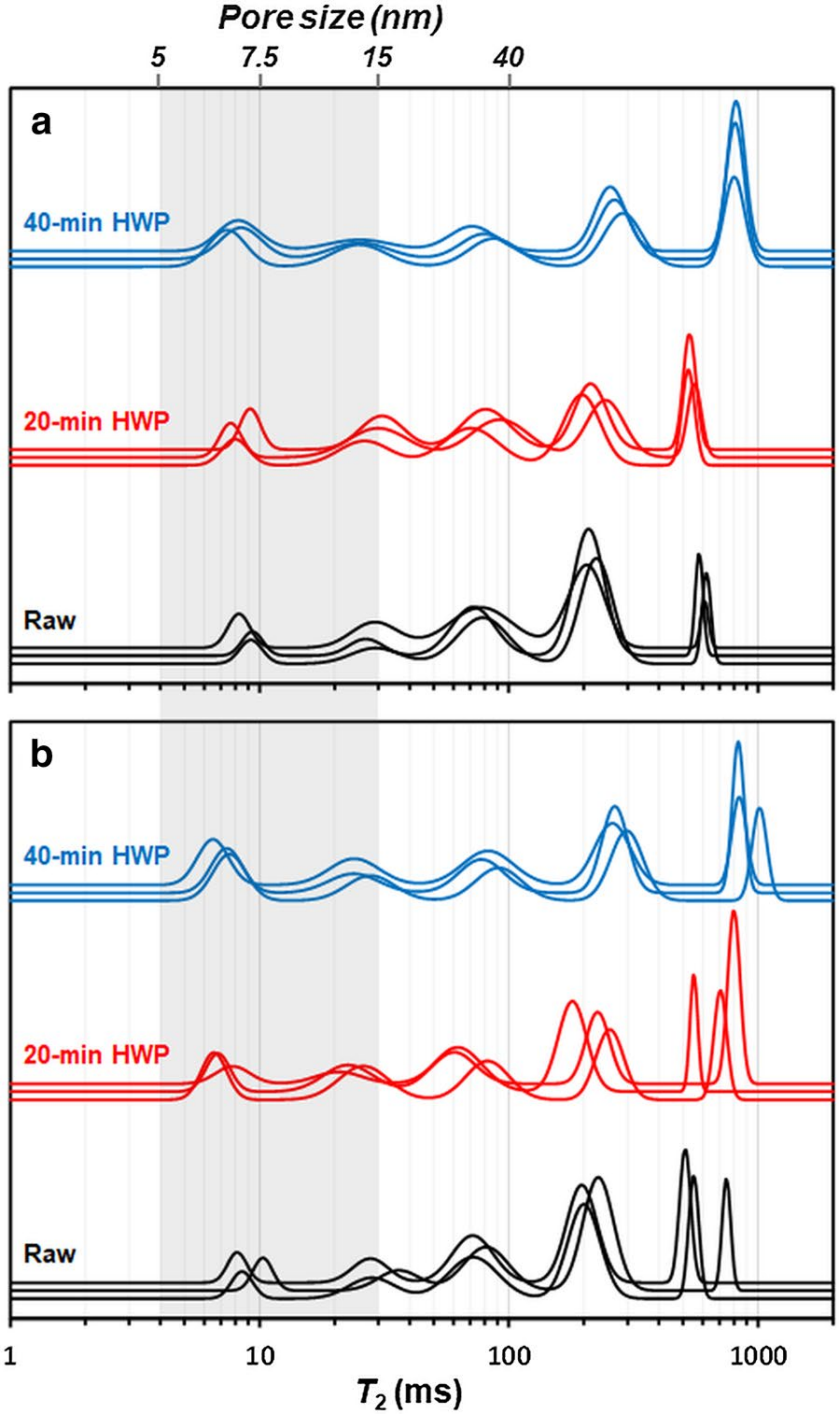
${T}_{2}$ distribution in **a** M7 and **b** M9 samples with 80% relative humidity (w/w). The x axis is the logarithmic scale of the relaxation time. The Y axis corresponds to the distribution amplitude expressed in normalized relative water content (% w/w). The correspondence between the relaxation time (${T}_{2}$) and the meso-porosity is indicated at the top of the figure. The experiments were realized in duplicate. The grey area, between 5 and 15 nm, corresponds to the range of pore size available for enzymes, the replicates are represented in the same color

Identifying changes in water mobility after HWP advances our understanding of the effect of HWP on enzymatic hydrolysis. The changes in water mobility were identified by NMR analyses of the relaxation times of water molecules in the CWR, which may be associated with a range of pore sizes, as previously reported [33, 34, 42, 83–85]. The NMR analyses were carried out under controlled relative humidity: at 20% (w/w) (Fig. 5), similar to the measurements performed in solid state NMR, and at 80% (w/w) (Fig. 6), similar to the humidity conditions which prevail during enzymatic hydrolysis.

At 20% humidity, two populations of water were detected. The HWP caused a decrease in peak width (PW) in both genotypes, indicating homogenisation of the water constraint within the CWR after the HWP (Additional file 3). It is worth noting that the HWP reduced the amplitude of the water populations at around 15 ms, which also suggests homogenisation of the CWR after pretreatment caused by domination of the main population. In the raw M9 CWR, the proportion of the water population (${P}_{2}$) associated with ${T}_{2}$ around 19 ms was higher than in M7, showing higher water mobility disparity in M9 genotype.

Further, the relaxation times of the different CWR were analysed at a relative humidity of 80% (w/w) to obtain more information on the effect of the HWP on water redistribution and porosity (Fig. 6). An increase in the number of populations with higher ${T}_{2}$ values was detected at 80% than at 20% relative humidity (2 populations with 20% versus 5 populations with 80% relative humidity). These populations represent distinct environments, with a different impact on the capacity of interaction and mobility of water molecules and probably resulting from the swelling of the cell wall caused by humidity. The water populations were divided into three regions and linked to different pore size ranges according to their ${T}_{2}$ values [34].

The first region of relaxation time from 4 to 30 ms (Fig. 6) corresponds to “small” pores between 5 and 15 nm in size (shaded area). This pore size range corresponds to the average enzyme size range [86, 87] and provides more information on the mobility capacity of the enzymes within the lignocellulosic matrix after HWP. Within this first region, there was an increase in the water content of the population (${P}_{2}$) with a ${T}_{2}$ of about 8.4 ms after HWP from 7.6 and 7.7% in the raw CWR to 16.8% and 18.3% with the 40-min HWP for CWR M7 and M9, respectively (Additional file 4). This increase can be explained either by the creation of new pores or by the compaction of larger domains during the HWP. The pretreatment also led to an increase in the inhomogeneity of this water content, visible through an increase in peak width (PW) after the 40-min HWP (Additional file 4). The water content associated with this range of pore sizes was highly correlated with the changes in different chemical parameters including hemicellulose loss (*R*^2^ = 0.98), Klason lignin content (*R*^2^ = 0.98), β–O–4 linkages estimation (*R*^2^ = − 0.93), and the molecular order (*R*^2^ = 0.96). These results highlight the fact that both the loss of hemicelluloses and changes in the ultrastructure of lignin have a direct influence on the porosity of the CWR, promoting the accessibility and circulation of water after HWP.

In the second region of ${T}_{2}$ from 30 to 100 ms, corresponding to pore sizes ranging between 15 and 40 nm, a decrease in the water content in this range of pore sizes was detected in both genotypes. This observation can be explained by the redistribution of water by diffusion to smaller size ranges. Indeed, the total water proportion (${P}_{2}$) in the third region, with a ${T}_{2}$ greater than 100 ms, remained stable after HWP. In this region, the mean ${T}_{2}$ of the two water pool environments increased with HWP from 210 to 271 ms and from 610 to 851 ms, respectively. Further, a transfer was observed from the water population with a ${T}_{2}$ of about 210 ms to the water population with a ${T}_{2}$ of about 600 ms. These observations suggest that HWP causes widening in the large domains.

The HWP modulated the domains and created new domains, where the water is more constrained, and which can be linked with pore sizes ranging from 5 to 15 nm. This increase in porosity, rendered visible by NMR and Simons’ staining, should favour the mobility of enzymes and hence facilitate their catalytic activity [33, 85].

### Correlation of physico-chemical modifications induced by HWP on saccharification

**Fig. 7:**
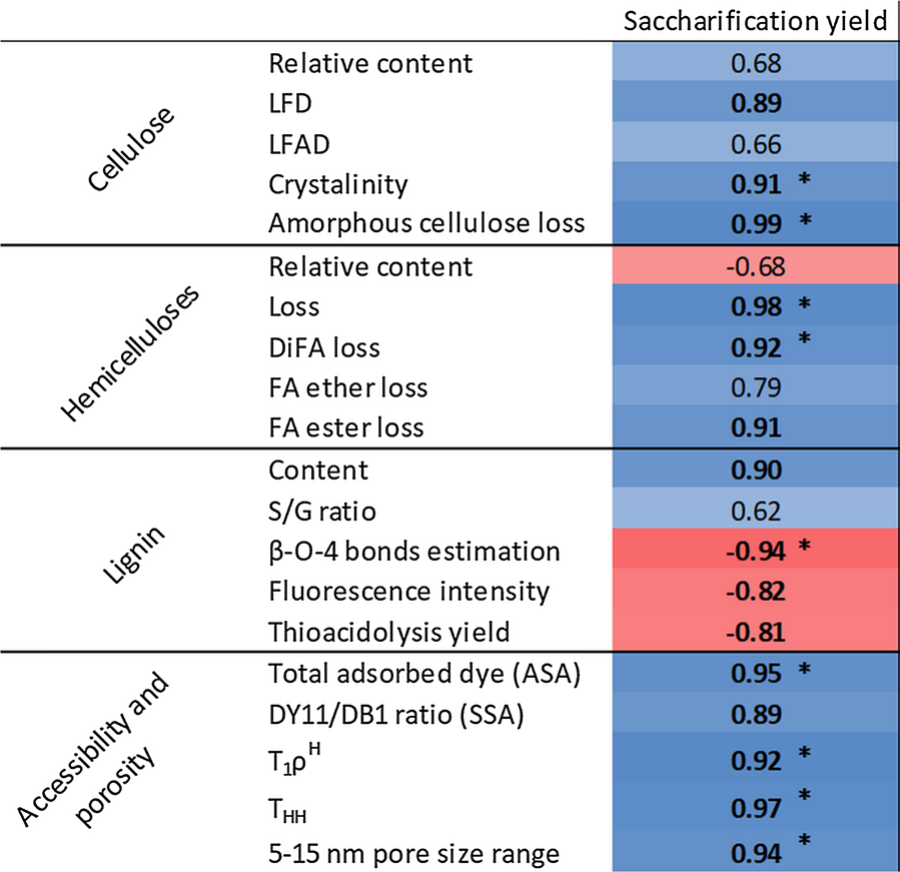
Pearson’s correlation coefficients between variations in physico-chemical factors and hydrolysis yield after 72 h. The red squares correspond to negative correlations and the blue squares to positive correlations. Values in bold are significant with a *p* values ≤ 0.05 and * have a *p* values ≤ 0.01

To measure the impact of the physico-chemical changes in enzymatic hydrolysis caused by the pretreatment, and to identify the main factors that contribute to recalcitrance, correlation coefficients were calculated between the saccharification yields at 72 h and the previously determined factors (Fig. 7).

HWP increased the porosity of the cell wall, as indicated by the increase in the proportion of the water pool associated with pore diameter in the range 5–15 nm, which includes the average diameter of enzymes involved in hydrolysis. The increase in this pore size range was significantly correlated with higher saccharification yield (*R*^2^ = 0.97), in agreement with the fact that 90% of the hydrolytic enzyme accessibility depends on the internal wall pores [9]. The increase in cell wall porosity was also supported by Simons’ staining as shown by the increased adsorption of DY11 probe, which has a *r*_H_ close to that of cellulase. The accessibility of cellulose estimated through ASA and more particularly for enzymes through SSA was closely correlated with the hydrolysis capacity of the CWR (*R*^2^ = 0.92).

This increase in porosity and in access to cellulose was achieved by modifying the intra- and inter-polymers interactions after the HWP. The analysis of cellulose interactions by solid-state NMR showed that HWP led to an increase in the molecular order surrounding the cellulose microfibrils (${T}_{1\rho }^{H}$) (*R*^2^ = 0.92) thanks to the decrease in the interactions between cellulose and amorphous components, such as hemicelluloses. In turn, this increase in the molecular order led to a loosening of the matrix and favoured the circulation/interaction of water molecules (${T}_{HH}$) (*R*^2^ = 0.97) and subsequently of enzymes. The loosening of the matrix surrounding the cellulose microfibrils was partly due to the breaking of the LCC [88], by reducing the bonds involving FA units, the major cross-linking agents in grasses [89]. In the present study, the loss of DiFA, which binds GAX together, was strongly correlated with saccharification yield (*R*^2^ = 0.96), in agreement with results reported in the literature on grass species, such as sugarcane [90], *Phalaris aquatica* and *Lolium* [91]. Interestingly, the loss of etherified FA was not strongly correlated with saccharification yield (*R*^2^ = 0.79), suggesting the destructuring of the GAX–GAX network is more important than that of GAX–lignin in enabling access by enzymes.

In addition to the role of hemicelluloses in the tight entanglement of the matrix, hemicelluloses also play a protective role by covering cellulose microfibrils with multiple layers and inserting themselves between them [92]. Indeed, hemicelluloses are bound to the hydrophilic surfaces of cellulose through hydrogen bonds [93], thereby reducing access to the cellulose to cellulases [36]. Thus, the loss of hemicelluloses induced by HWP helped increase accessibility of the enzyme within the matrix and also of the SSA, as shown by the strong correlation between hemicellulose loss and saccharification yield (*R*^2^ = 0.98). During HWP, the loss of hemicelluloses also led to an increase in lignin content. Indeed, it has been shown that the presence of lignin reduces the enzymatic hydrolysis potential through its role as a physical barrier, specifically, through its interactions with polysaccharides that reduce the accessibility of cellulose, and its non-specific interaction capacity with hydrolytic enzymes [7, 94, 95]. After HWP, the increase in lignin content was positively correlated with the saccharification yield (*R*^2^ = 0.90), suggesting that the lignin content was not directly linked to the recalcitrance of lignocellulose, as already demonstrated in previous studies [57, 96, 97]. Thus, chemical pretreatments aimed at eliminating lignin, such as oxidative alkaline or organosolv pretreatments, are not necessarily the best ways to improve saccharification [33, 97]. Considering lignin organisation is a more suitable way to assess its impact on recalcitrance than lignin content alone.

Our investigations revealed that changes in the concentration of polymers and their interactions during the HWP were associated with several changes in their ultrastructure, more particularly that of lignin. First, HWP has been shown to increase the S/G ratio but in the present study the ratio was not significantly correlated with hydrolysis capacity (*R*^2^ = 0.62), as already shown [98]. This shows that the S/G ratio determined by thioacidolysis cannot predict hydrolysis capacity [99], its impact is not yet clear and depends on the LB species considered. The decrease in the estimated ether bonds (mainly β–O–4) as well as the increase in the condensation state reflected by the thioacidolysis yield were both negatively correlated with saccharification yield (*R*^2^ = − 0.94 and − 0.81, respectively). These results suggest that the condensed state of lignin after HWP may promote enzymatic deconstruction. This correlation has already been observed [66, 98, 100] and may be explained by the variation in the lignin structure according to its inter-unit bonds [101]. Indeed, molecular modelling has shown that a high concentration of β–O–4 bonds extends lignin structure thereby favouring interactions with cellulose fibril thereby forming a physical barrier and limiting access to cellulose by cellulases [101]. The changes in the structure of lignin caused by HWP are hypothesised to reduce its protective role and to facilitate access to the cellulose microfibrils by cellulases [102]. Conversely, excessive condensation of lignin could be detrimental to enzymatic hydrolysis as it would promote strong aggregation of cellulose and, therefore, reduce its accessibility for cellulases [76]. This may be the case for the M9 genotype after the 40-min HWP, as strong aggregation of cellulose microfibrils was observed together with a lower saccharification rate than for the M7 genotype after the 40-min HWP. This means that severity of the HWP needs to be balanced to enable sufficient lignin condensation to facilitate access to cellulose while simultaneously limiting strong interactions. Fast and easy prediction of saccharification based on lignin organisation seems possible by measuring CWR autofluorescence (*R*^2^ = − 0.82), as previously reported with other pretreatments and LB species [33, 58]. It was previously assumed [103] that lignin does not have a direct impact on the accessibility of enzymes but has an indirect effect on hydrolysis capacity through its strong interaction with hemicelluloses. Our study confirms that reshaped organisation of lignin in the cell wall after HWP does have a direct impact on access to cellulose and on the saccharification potential.

HWP also had an impact on the ultrastructure of the cellulose by increasing its crystallinity and LFD. Crystallinity is often described as a limiting factor in enzymatic hydrolysis [7, 104, 105]. In the present study, the increase in the crystallinity of the cellulose resulting from the loss of more hydrolysable amorphous cellulose during HWP, had no negative impact on the saccharification capacity of the CWR (*R*^2^ = 0.91). The increase in LFD also had a positive impact on saccharification (*R*^2^ = 0.89). This observation could be linked to swelling of the cellulose due to the opening of the matrix, leading to an increase in SSA.

## Conclusion

Our work has shown that hot water pretreatment (HWP) improves enzymatic hydrolysis by altering many interconnected factors ranging from the polymers' ultrastructure (bounds, crystallinity) to the organisation of the wall (mesoporosity). The loss of hemicelluloses and changes in the structural features of the polymers induced by HWP leads to significant reorganization of the lignocellulosic matrix. These changes result in an increase in the specific surface area of the cellulose and redistribution of water thereby improving accessibility to cellulose by cellulases and enhancing hydrolysis. Interestingly, the environment and organization of lignin is more important than its concentration in explaining cellulose accessibility.

The multiscale combination of biochemical compositional techniques with solid-state NMR and NMR relaxometry analysis was decisive in allowing us to understand the interplay between polymers and the recalcitrance of hot water pretreated LB. The same approach could be applied to other biomass species and other pretreatments to identify the best compromise between the severity of the pretreatment and saccharification by elucidating the interactions between polymers.

## Materials and methods

### Plant materials

Two maize genotypes F7025 (abbreviated as M7) and F98902 (as M9) were selected based on the results of preliminary composition experiments indicating differences in digestibility capacity, high and low digestibility, respectively [106, 107]. The genotypes were cultivated in INRAE experimental plots in Mauguio (South of France) under irrigated conditions and harvested at the silage stage. The internode under the main ear was isolated, dried and cut into 2-cm fragments. The cell wall residue (CWR) was obtained after removal of soluble components in an 8-h ethanol extraction followed by a 48-h water extraction.

### Hot water pretreatment

Hot water pretreatment (HWP) was performed on 2-cm half-fragments of maize CWR in mineralization bombs equipped with Teflon cups (Parr, USA). Each half-fragment was pretreated with a volume of deionized water adjusted to a ratio of 1:30 (v/w) [33], in an oil bath maintained at a constant temperature of 180 °C during two treatment durations (20 and 40 min). CWR were cooled in ice and then washed with 50% ethanol solution and deionized water until the soluble components were completely removed. The pretreatment parameters were chosen based on the results of previous experiments to preserve the structure of the CWR for microscopy imaging [33, 41, 108].

The consolidated severity factor (CSF) of these pretreatments was calculated according to the following equation [109]:

1 $$ \text{CSF} = \text{log} \left[ { t \times \text{exp} \left( {\frac{{T_{i} - T_{ref} }}{14.75}} \right) } \right], $$

where *t* is the time of pretreatment, ${T}_{i}$ the temperature of the pretreatment (180 °C) and ${T}_{ref}$ the reference temperature of 100 °C.

### Compositional analyses of CWR

For the biochemical and physico-chemical analysis, the CWR were reduced to an average granulometry of 1 mm with an ultra-centrifugal mill (ZM200, Retsch, Germany).

#### Polysaccharide analysis

The concentrations of monomeric sugars were quantified by HPAEC-PAD after two-step H_2_SO_4_ hydrolysis as described previously [110].

#### Ferulic acid and p-coumaric acid content

##### Mild and severe alkaline hydrolysis

The esterified and etherified contents of ferulic acid and *p*-coumaric acid were determined by alkaline hydrolysis at two different severities. Mild alkaline hydrolysis was applied on 10 mg of CWR with 1 mL of 1 M NaOH for 16 h at room temperature. Severe alkaline hydrolysis was applied on 10 mg of powder with 2 mL of 4 M NaOH for 2 h at 170 °C. The hydrolysate was subsequently acidified to pH < 3 (with 6 M HCl), centrifuged at 12,000*g* for 10 min, purified in on a solid-phase extraction cartridge (Waters Sep-pack t18, USA) with 0.1% HCOOH and the phenolic compounds were then eluted with methanol. The phenolic compounds were analysed by HPLC as detailed in [111].

Ferulic acid and *p*-coumaric acid were quantified using *o*-coumaric acid as an internal standard and *o*-coumaric acid, ferulic acid and *p*-coumaric acid as standard solution.

The concentration of esterified hydroxycinnamic acids was determined by the amount of ferulic and *p*-coumaric acid released during mild alkaline hydrolysis. Etherified ferulic acid content was calculated as the difference between the total amount of esterified ferulic acid released by mild and the total amount of ferulic acid released by severe alkaline hydrolysis.

#### Acetyl content

Alkaline hydrolysis was performed on 5 mg of CWR with 1 M NaOH at 4 °C for 30 min. After stabilization of the pH at 8 with 1 M HCl, acetyl content was quantified with a commercial acetic acid kit (BiosenTec, Portet-sur-Garonne, France).

#### Klason lignin content

Aliquots (200 mg) of CWR (M) were hydrolysed with 2 mL of 12 M sulphuric acid solution at room temperature for 2 h. The hydrolysate was diluted with deionized water to bring the concentration of sulphuric acid to 2 M and the mixture was incubated for 3 h at 300 °C with agitation of 500 rpm. The solid residues were filtered and washed with deionised water, dried at 100° C for 20 h and then weighed (${W}_{1}$). Finally, the sample was calcined at 600 °C for 3.5 h and weighed again (${W}_{2}$) [112].

The total Klason lignin content was calculated according to the following equation:

2 $$ KL\,\left( \% \right) = \frac{{\left( {W_{1} - W_{2} } \right)}}{M} \times 100. $$

#### Thioacidolysis

The monomeric composition of the ether-linked lignin fraction was determined by thioacidolysis as described by [113]. The reaction reagent composed of dioxane/boron trifluoride etherate/ethanethiol (87.5/2.5/10, v/v/v) was prepared and 1 mL was added to 10 mg of CWR with 1 mL of internal standard tetracosane at 0.25 mg mL^−1^, and the mixture was then heated at 100 °C for 4 h. The phenolic compounds were extracted from the reaction medium by adding 3 × 25 mL of dichloromethane and concentrated by evaporation. The monomeric units of the lignin were then silylated for quantification in a gas chromotographer equipped with a fused silica capillary DB1 column (30 m × 0.3 mm) (J&W Scientific, USA) and a flame ionisation detector.

#### FTIR analysis

Pellets were prepared from 2 mg of CWR in powder form mixed with 300 mg of KBr, then analysed by MIR on a Nicolet 6700 Thermo Electron FTIR spectrometer with a KBr separator and a DTGS KBr detector. Spectra were recorded three times in the range 4000–400 cm^−1^ with a resolution of 4 cm^−1^ and 16 scans per sample. The baseline of the spectra was corrected using Omnic software and then normalized by area by applying a correction factor of $\frac{1000}{{A}_{2000-800 cm-1}}$, where ${A}_{2000-800}$ cm^−1^ is the area of the spectra between 2000 and 800 cm^−1^.

#### 3D fluorescence maps

3D fluorescence maps of raw and hot-water pretreated CWR were acquired using a JASCO FP8300 spectrofluorometer (Japan). The 3D maps were acquired with excitation wavelengths scanning from 300 to 550 nm, with a wavelength increment of 2 nm, emission wavelengths scanning from 350 to 600 nm, with wavelength increment of 0.2 nm. The sensitivity was set to 500 V. The 3D maps were analysed with Jasco SpectraManager software.

### Simons’ staining

The accessible surface area of the cellulose and the relative porosity of the LB were determined by Simons’ staining as previously described [114, 115]. Direct Blue 1 (DB1) was purchased from Pylam Products Company (USA). Direct Yellow 11 (DY11) was obtained from Sigma-Aldrich (USA). DY11 was purified with 100 kDa (molecular weight cutoff) polyethersulfone membranes, and only the supernatant with a MW > 100 kDa, was used. DB1 and DY11 were prepared to yield 10 mg/mL by adding deionised water. Substrate (10 mg) was added in 2-mL tubes and mixed with 0.1 mL of phosphate buffer (140 mM NaCl, 0.3 M phosphate, pH 6.0), an increasing volume of both dyes (0.025, 0.05, 0.75, 0.1, 0.15 and 0.2 mL) followed by the addition of deionized water to bring the final volume to 1 mL. The tubes were incubated at 70 °C for 6 h with agitation of 300 rpm. After incubation, the tubes were centrifuged at 12,000*g* for 5 min, and the absorbance of the supernatant was analysed after 100-fold dilution with a spectrophotometer (UV-3100 PC, VWR, USA) at 430 and 600 nm. The amount of dye adsorbed onto the biomass was determined as the difference between the concentration of the initially added dye and the concentration of the dye in the supernatant, as described by Alam et al. [81].

### ^13^C Solid-state NMR

Approximately 80 mg of each CWR were rehydrated to 24–26% (w/w) with ultra-pure water. All the analyses were performed as biological triplicates. The solid-state NMR spectra were recorded on a Bruker Avance III 400 MHz spectrometer operating at a carbon frequency of 100.62 MHz. A double resonance ^1^H/X CP/MAS 4 mm probe coupled with a high power amplifier was used for the ^13^C CP/MAS experiment. The magic angle spinning (MAS) rate was set at 12 kHz and each acquisition was acquired at ambient temperature (25 °C). The experiment was conducted under a 90° proton pulse of 2.6 ± 0.1 µs, a contact time of 1.5 ms and a 10 s recycling time. Each spectrum was the result of the accumulation of 2048 scans. Chemical shifts were calibrated using glycine as external reference, assigning the carbonyl at 176.03 ppm.

Chemical shifts, half width, and the peak area of the samples were determined using a least-squares fitting method with Peakfit® software (Systat Software Inc., USA).

According to the method of Larsson et al. [116], cellulose crystallinity was calculated from deconvolution of cellulose C_4_ peaks in the region of 80–91 ppm according to the following equation:

3 $$ \text{CrI} = \frac{\sum \,area \,88.1 \,to\, 86.2\, \text{ppm} }{{\sum \,area\, 88.1 \,to\, 82.9\, \text{ppm} }}. $$

This method uses three Lorentzian peaks corresponding to cellulose Cr(Iα) (88.1 ppm), cellulose Cr(Iα + β) (86.8 ppm) and cellulose Cr(Iβ) (86.2 ppm). An additional Gaussian peak associated with the para-crystalline (PCr) contribution (87.9 ppm) was also used. Three peaks were used in the amorphous C4 region, the two Gaussian peaks corresponding to the accessible surface cellulose C4 (82.9 and 84.1 ppm) and the other to the inaccessible surface C4 (83.4 ppm). The proportion of crystalline cellulose was determined by dividing the total peak area of four crystalline cellulose C_4_ peaks by those of seven cellulose C_4_ peaks. The lateral fibre dimension (LFD) and the lateral dimensions of the fibril aggregates (LFAD) were also estimated assuming the cross section of cellulose microfibre is square and all the amorphous cellulose is attached to the surface of the fibre. The width of the cellulosic chains was set at 0.57 nm [117].

The molecular dynamics of the samples was further characterised by varying the contact time (τ) from 10 to 9000 µs. Twenty CP/MAS spectra were recorded with an accumulation of 512 scans per contact time. The changes in carbon peak area (C_4_ of crystalline cellulose and O–CH_3_ of pectin methyl ester) between the different groups were fitted according to the following equation [118]:

4 $$ I\left( {\uptau } \right) = I_{0} e^{{ - {\uptau }/T_{{1{\uprho }}}^{H} }} *\left\{ {1 - {\uplambda }e^{{ - {\uptau }/T_{HH} }} - \left( {1 - {\uplambda }} \right)e^{{ - 3{\uptau }/T_{2HH} }} e^{{ - {\uptau }^{2} /2T_{CH}^{2} }} } \right\}, $$

where $I\left(\uptau \right)$ is the carbon peak area (C_4_ of crystalline cellulose and O–CH_3_ of pectin methyl ester) according to the contact time ($\uptau$), ${I}_{0}$ is the maximum carbon signal intensity (associated with the optimal contact time), λ is a parameter that depends on the number of protons (n) carried by carbons (λ = 1/(n + 1)), ${T}_{CH}$ is the mean dipolar coupling between carbon and covalently linked proton, ${T}_{1\rho }^{H}$ is the spin–lattice proton relaxation time in the rotating frame, ${T}_{HH}$ is the spin diffusion time between two nearby protons.

### Time-domain NMR

These interactions can be linked to the porosity of the system. Aliquots (80 mg) of each CWR were analysed at two hydration levels, at about 20% (low) and 80% (high) (w/w). Transverse relaxation (*T*_2_) was measured at 4 °C on a Bruker Minispec mq20 (0.47 T), equipped with a thermostated ^1^H probe, using the Carr–Purcell–Meiboom–Gill (CPMG) pulse sequence. The echo time for low hydration was 80 µs, 400 even echoes were collected, and 384 scans were acquired with a recycle delay of 3 s. The echo time for high hydration was 1 ms, 2000 even echoes were collected, and 128 scans were acquired with a recycle delay of 7 s. An inverse Laplace transformation (ILT) was applied [119] to convert the relaxation signal into a continuous distribution of the relaxation components [33].

### Enzymatic saccharification

Enzymatic saccharification was performed using the commercial enzyme cocktail Cellic CTec 2 (Novozymes, Denmark) containing cellulase and xylanase activity of 205 FPU.mL^−1^ and 9,068 IU mL^−1^, respectively. Raw and pretreated milled CWR (20 mg) were added to 1 mL of 50 mM acetate buffer pH 5.2 with 0.15 mg mL^−1^ of tetracycline as antibiotic, 0.04 mg mL^−1^ cycloheximide as antifungal agent, to 2 mL tubes. The micro-reactors (powder and medium) were pre-incubated for 20 min at 50 °C, the Cellic CTec 2 cocktail was then loaded at a final concentration of 30 FPU.g^−1^ of CWR and the mixtures were incubated at 50 °C with agitation of 300 rpm.

Aliquots (12 μL) were taken at different hydrolysis timepoints: 0, 0.5, 1, 2, 4, 8 h. The aliquots and final 72 h hydrolysate were heated at 100 °C for 2 min, and then centrifuged at 12,000*g* for 5 min. The kinetic of release of reducing sugars hydrolysed over time were determined via DNS assay and the final concentrations of neutral sugars hydrolysed after 72 h were determined by gas–liquid chromatography.

#### Determination of reducing sugars by DNS assay

The 3,5-Dinitrosalicylic acid (DNS) reagent was prepared according to the protocol established by [120]. In 1.5 mL tubes, 60 µL aliquots diluted 10 times were mixed with 120 µL of DNS and heated at 100 °C for 10 min. The reducing sugars in the aliquots were determined with a spectrophotometer (UV–3100 PC, VWR, USA) at 540 nm after diluting 100 μL of solution in 1.25 mL of deionized water. A standard curve of glucose was used to calculate the equivalent glucose reducing sugar in the different CWRs.

The percentage sugars converted during saccharification were calculated according to the following equation:

5 $$ {\text{Converted \,sugars }}\left( \% \right)\, = \,\left( {\frac{\%\, of \,released\, sugars}{{\%\, of \,sugars\, in\, the\, sample\, before\, hydrolysis}}} \right) \times 100. $$

The initial reaction rate was calculated as the tangent to the hydrolysis curve, converted sugars (g L^−1^) plotted against reaction time (hours), at time 0, and is expressed in g L^−1^ h^−1^.

#### Quantification of neutral sugars by gas–liquid chromatography

Quantification of neutral sugars were performed by gas–liquid chromatography on a TG-225 GC Column (30 × 0.32 mm ID) using a TRACE™ Ultra Gas Chromatograph (Thermo Scientific™; column temperature: 205 °C; split injector temperature: 220 °C; flame ionization detector temperature: 250 °C; carrier gas: H^2^) after a single sulfuric acid 2N degradation and derivatization as alditol acetates according to [121].

### Correlation analysis

Pearson’s simple correlation coefficient (*R*^2^) and Pearson’s correlation matrix were calculated with SigmaPlot 12.0 (Systat Software Inc., USA). According to the population size, for pairs of variables with *p* values lower than 0.050, there was a significant relationship between the two variables.

## Supplementary Information

**Additional file 1: Fig. S1.** Kinetics of the release of monosaccharides during saccharification of raw and HWP samples**.** A) M7 samples, B) M9 samples.

**Additional file 2: Fig. S2.** Pearson’s correlation matrix calculated between two variables. The red squares correspond to negative correlations and the blue squares to positive correlations. Values in bold are significant (*p* values ≤ 0.05).

**Additional file 3: Table S1.** Relaxation time (${T}_{2}$) values, water proportion (${P}_{2}$) and pick width proportion (PW%) of the peaks represented in the Fig. 5. The PW was normalized to the ${T}_{2}$ value. Results are expressed as means of 3 repetitions with standard deviation into parenthesis.

**Additional file 4: Table S2.** Relaxation time (${T}_{2}$) values, water proportion (${P}_{2}$) and pick width proportion (PW%) of the peaks represented in the Fig. 6. The PW was normalized to the ${T}_{2}$ value. Results are expressed as means of 3 repetitions with standard deviation into parenthesis.

## Data Availability

All data generated or analysed during this study are included in this published article and its supplementary information files.

## Abbreviations

ASA: Accessible surface area
CP/MAS NMR: Cross polarization/magic angle spinning nuclear magnetic resonance
CPMG: Carr-Purcell-Meiboom-Gill
Cr: Crystalline region of cellulose
CSF: Consolidated severity factor
CWR: Cell wall residue
DB1: Direct blue 1
DiFAe: Esterified diferulic acid
DNS: 3,5-Dinitrosalicylic acid
DY11: Direct yellow 11
FA: Ferulic acid
FTIR: Fourier-transform infrared spectroscopy
GAX: Glucuronoarabinoxylans
HPAEC-PAD: High-performance anion-exchange chromatography with pulsed amperometric detection
HWP: Hot water pretreatment
ILT: Inverse Laplace transformation
KL: Klason lignin
LB: Lignocellulosic biomass
LCC: Lignin-carbohydrate complex
LFD: Lateral fibre dimension
LFAD: Lateral fibril aggregates dimension
M7: F7025 genotype
M9: F98902 genotype
$${P}_{2}$$: Peak proportion;
p-CA: p-coumaric acid
PCr: Para-cristalline region of cellulose
PW: Peak width
*R*^2^: Pearson’s simple correlation coefficient
*r*_H_: Hydrodynamic radii
S/G: Syringyl/guaiacyl lignin unit
SSA: Specific surface area
${T}_{2}$: Transverse relaxation time
${T}_{1\rho }^{H}$: Spin-lattice proton relaxation time in the rotating frame
${T}_{HH}$: Spin diffusion time between two nearby protons
VCT: Variable contact time
