# Supplementary figures and images for: Evaluating polymer interplay after hot water pretreatment to investigate maize stem internode recalcitrance
Source: Biotechnol Biofuels. 2021 Jul 31;14:164. doi: 10.1186/s13068-021-02015-8 (PMC8325808; doi:10.1186/s13068-021-02015-8)

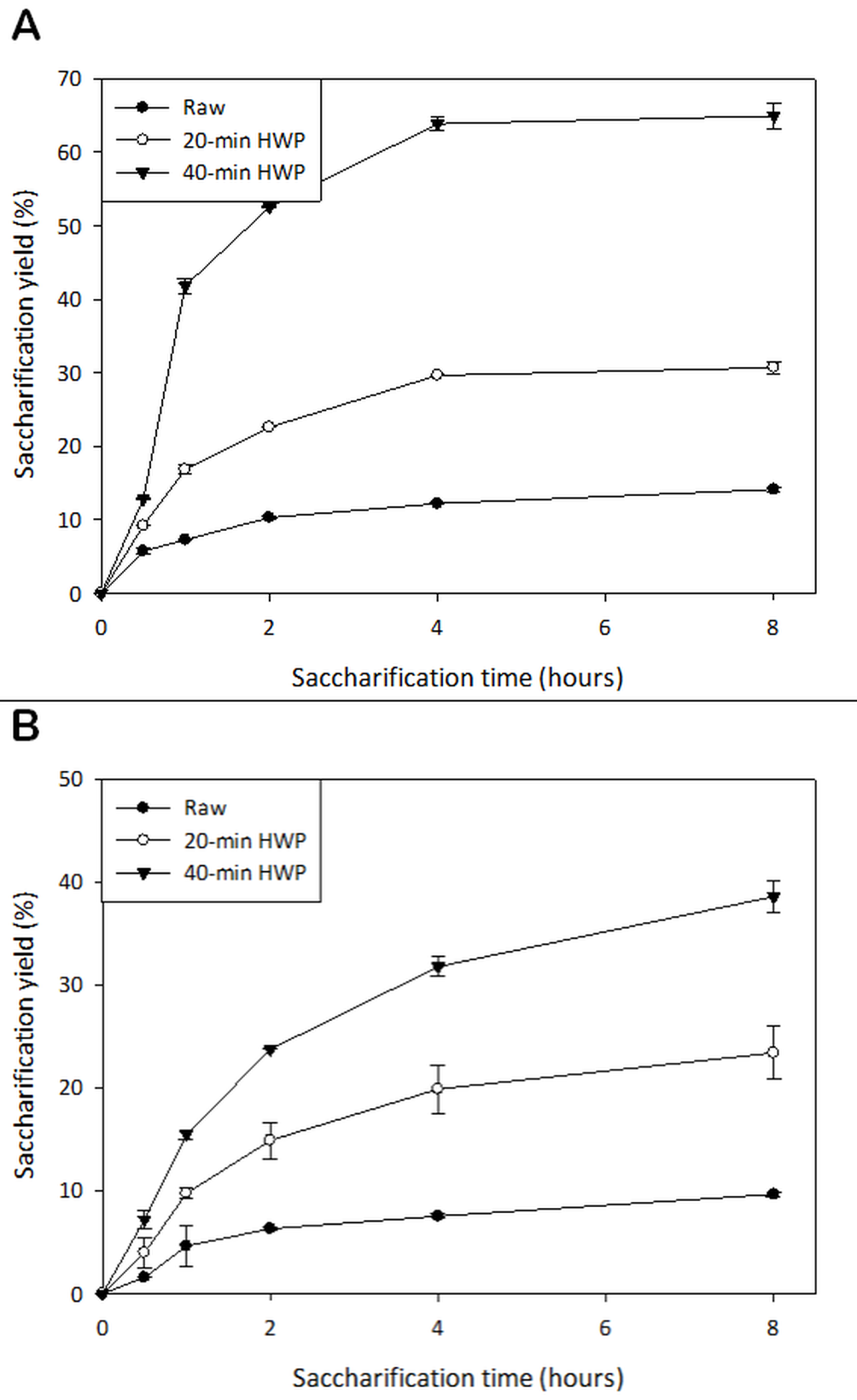

Supplement: Supplementary file 1 — Additional file 1: Fig. S1. Kinetics of the release of monosaccharides during saccharification of raw and HWP samples. A) M7 samples, B) M9 samples. [file 13068_2021_2015_MOESM1_ESM.tif]

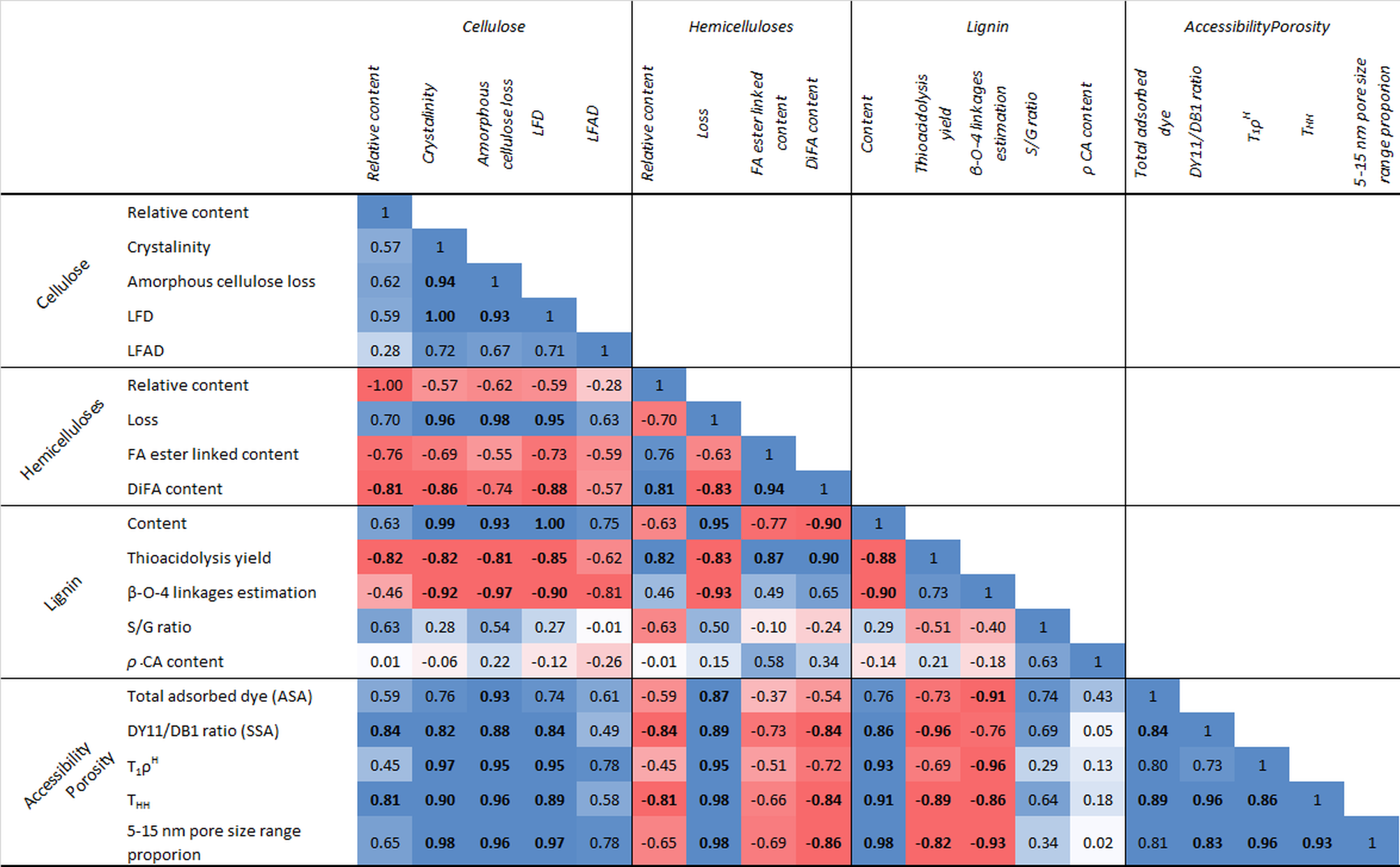

Supplement: Supplementary file 2 — Additional file 2: Fig. S2. Pearson’s correlation matrix calculated between two variables. The red squares correspond to negative correlations and the blue squares to positive correlations. Values in bold are significant (p values ≤ 0.05). [file 13068_2021_2015_MOESM2_ESM.tif]
